# Development and psychometric properties of the critical thinking attitude scale in Italian college students

**DOI:** 10.3389/fpsyg.2025.1599920

**Published:** 2025-10-07

**Authors:** Rosa Angela Fabio, Alessandro Antonietti, Paola Iannello, Rossella Suriano

**Affiliations:** ^1^Department COSPECS, University of Messina, Messina, Italy; ^2^Department of Psychology, Catholic University of the Sacred Heart, Milan, Italy

**Keywords:** critical thinking, critical thinking attitude, measurement tool, psychometric properties, validation

## Abstract

**Introduction:**

Critical thinking (CT) is a higher-order cognitive skill essential for academic and professional success, combining the ability to evaluate evidence with a reflective and analytical attitude toward information. Although several tools exist to measure critical thinking disposition, many show limitations in length, reliability, or generalizability, often focusing on specific populations such as medical and nursing students. This study addresses this gap by developing and validating the Critical Thinking Attitude Scale (CTAS) for Italian university students.

**Methods:**

The study was conducted in two phases. In Study 1, 211 participants completed the CTAS questionnaire; exploratory factor analysis revealed a four-factor structure: systematicity, search for truth and openness, analyticity, and inquisitiveness, with high internal consistency (α > 0.78). In Study 2, involving 577 participants, the four-factor model was confirmed through confirmatory factor analysis, showing excellent reliability (α = 0.93) and significant correlations with the Critical Reasoning Assessment (CRA) and the Italian Big Five Inventory (BFI-10), supporting convergent validity, while negative correlations with the Dysfunctional Beliefs Questionnaire (DBQ) highlighted divergent validity.

**Results:**

Exploratory and confirmatory analyses supported a robust four-factor structure of the CTAS, demonstrating excellent internal consistency and reliability. Convergent validity was confirmed through positive correlations with CRA and BFI-10, whereas divergent validity was supported by negative correlations with DBQ.

**Discussion:**

This study fills an instrument gap by providing the first Italian adaptation of the CTAS, offering a psychometrically robust and culturally appropriate tool for assessing attitudes toward critical thinking across disciplines. The findings provide a foundation for targeted educational interventions and future research aimed at fostering reflective and analytical thinking in higher education, contributing a standardized, valid, and broadly applicable measure.

## Introduction

Critical thinking (CT) refers to higher-order cognitive skills involving the critical evaluation of evidence, the ability to recognize and correct reasoning errors, and a propensity to consider different perspectives (Zwiers and Crawford, 2023). This competence translates into a reflective approach to forming judgments and constructing valid arguments based on thoughtful reasoning (Hwang et al., 2023). Critical thinking is crucial in the context of the complex challenges of contemporary society, contributing to success in both learning and professional careers (Sk and Halder, 2024; Suriano et al., 2025; Fabio and Suriano 2021). In educational settings, it contributes to academic success by enabling individuals to critically analyse complex information, thoroughly understand concepts, and creatively solve problems. These skills not only facilitate excellence in formal learning but also prepare individuals to make informed decisions and tackle complex professional challenges (Teng and Yue, 2023). In academic settings, university students who think critically can analyse complex situations, evaluate options, and make well-considered decisions, often demonstrating greater effectiveness and success in managing challenges. The ability to think critically allows them to approach problems innovatively and adapt to rapidly changing environments (Fabio and Suriano, 2023, 2025).

Thinking critically involves both high-level abilities and an attitude toward critical thinking (Saltor et al., 2023). Ability is demonstrated through the actual skill in applying critical thinking through specific actions and cognitive processes. Attitude represents the inclination or mental disposition that drives an individual to evaluate information, arguments, or situations rather objectively, analytically, and reflectively than accepting them uncritically. An attitude toward critical thinking guides individuals in the effective application of critical thinking skills and is closely connected to the ability for critical thinking (Zhang et al., 2023). An individual with a strong attitude toward critical thinking will be inclined to question critically, challenge received information and seek a deeper understanding. This attitude fosters the willingness to examine different perspectives, tackle the challenge of ambiguity, and resist uncritically accepting information (Kaczkó and Ostendorf, 2023).

There are several theories that attempt to elucidate the construct of critical thinking. The Reflective Judgment Model by King and Kitchener (2004) proposes an evolutionary description of critical thinking ability divided into three sequential stages. The pre-reflective stage highlights dependence on direct sources without critical evaluation, followed by the quasi-reflective stage, characterized by increased awareness of uncertainty. In the reflective stage, uncertainty is accepted, and a clear decision-making process based on critical evaluation of available evidence is adopted (King and Kitchener, 2004). Another model suitable for explaining critical thinking is the dual-process model (Evans and Stanovich, 2013), which emphasizes the presence of two distinct reasoning systems. System 1 operates through automatic and intuitive mental processes guided by information processing heuristics. In these circumstances, decisions and judgments are formulated instantly without the individual’s conscious involvement, drawing on past knowledge and beliefs. On the other hand, System 2 operates through an analytical information processing process, requiring deliberate control by the individual. Although slower and more resource-intensive, it allows for a more logical evaluation of information, aligned with the tenets of critical thinking theory.

### The importance of measuring critical thinking attitude in college students

The assessment of critical thinking attitude in university students has significant implications for their academic and professional development (Sk and Halder, 2024). In healthcare and other fields, critical thinking is essential for making informed decisions based on complex data (Hwang et al., 2023; İlaslan et al., 2023). Despite the predominant focus on evaluating this competence among nursing and medical students, it is crucial to recognize the importance of critical thinking in other fields of knowledge as well. In the field of economics, critical thinking is essential for understanding and analyzing complex economic phenomena, developing theoretical models, and applying them to practical realities (Anum and Olumuyiwa, 2024). Economics students must be able to critically evaluate fiscal and monetary policies, analyze statistical data, and comprehend market dynamics (Azzaakiyyah et al., 2023). This competence is crucial for formulating accurate economic forecasts, making informed decisions, and solving complex economic problems. In the social sciences, critical thinking skills are fundamental for evaluating sociological theories, analyzing statistical data, and understanding social phenomena through rigorous methodological approaches (Vincent-Lancrin, 2023). In psychology, critical thinking is essential for interpreting experimental research, applying theoretical concepts to practical situations, and developing evidence-based interventions (Silva et al., 2023). The humanities disciplines, such as philosophy and literature, require students to exercise critical thinking in analyzing complex texts, developing coherent arguments, and reflecting on ethical and moral issues (Baddane and Ennam, 2024). These skills are fundamental in shaping individuals capable of addressing intellectual and social challenges in a reflective and articulate manner. Critical thinking also plays a central role in the natural sciences and engineering disciplines (Cossu et al., 2024). Students must be able to design and conduct experiments, interpret scientific results, and solve technical problems with creativity and analytical rigor. The ability to critically evaluate their own hypotheses and results is essential for the advancement of scientific and technological knowledge (Karaer et al., 2024). Therefore, it is useful to develop and implement critical thinking assessment tools that are applicable across all academic disciplines.

### Measuring critical thinking attitude

Several tools have been used to assess critical thinking (CT), but as highlighted by Quinn et al. (2020), few have specifically measured the construct of critical thinking attitude. Measuring CT attitude can be crucial in identifying targeted goals to promote CT skills through focused educational programs.

One well-known and widely used tool is the California Critical Thinking Disposition Inventory (CCTDI), developed by Facione et al. (1994) based on the consensus statement from the Delphi study on the critical thinker profile. Designed to assess CT disposition in the general adult population across various contexts, the CCTDI consists of 75 items divided into seven subscales: truth-seeking, open-mindedness, analyticity, systematicity, curiosity, self-confidence, and maturity. Specifically, truth-seeking reflects an individual’s aspiration to pursue accurate and in-depth knowledge in every field. Open-mindedness is manifested through tolerance for diverse perspectives and awareness of one’s own biases. Analyticity implies reasoning based on concrete evidence and the ability to tackle conceptual or practical challenges. Systematicity implies an organized, focused, and diligent approach to inquiry. Curiosity is the intrinsic desire to explore and learn. Self-confidence pertains to assurance and belief in one’s own reasoning processes. Lastly, maturity is associated with the ability to make wise decisions. Over time, the tool has undergone various revisions and has been translated into many languages (Bravo et al., 2020; Cui et al., 2021; Nguyen et al., 2023). Despite its extensive use within the scientific community, the literature has highlighted uncertainties regarding the consistency of preliminary results regarding the reliability and validity of its original version. Although the overall reliability of the California Critical Thinking Disposition Inventory (CCTDI) may be acceptable in its original form, the reported Cronbach’s alpha values for the initial pilot testing of the seven CCTDI scales are moderate, ranging from 0.71 to 0.80; however, those of the subscales appear relatively low. Other research conducted on the CCTDI has reported variable results. For example, in Chinese students, lower alpha coefficients ranging from 0.59 to 0.75 have been observed (Zhang, 2003). In the case of Taiwanese and North American students, Yeh (2002) reported alpha values ranging from 0.34 to 0.73 in the Chinese version and values ranging from 0.52 to 0.73 in the English version, with an overall alpha of 0.71 in both cases. Additionally, factorial analyses from various studies have suggested modifications in dimension combinations and a reduction in the number of items in many of these studies.

Therefore, partial versions of the CCTDI have been developed, which in some cases have demonstrated greater reliability compared to the original scale. Some eliminated items may not have effectively measured due to participant fatigue, suggesting that the length of the original scale could represent a burdensome task for subjects (Bowling et al., 2022). Hwang et al. (2010) and Wang et al. (2019) addressed this issue by reducing the number of items from 75 to 18 through exploratory factor analysis (EFA). A shorter scale encourages participants to complete the survey and pay more attention to each question, facilitating more thoughtful and accurate responses (Hwang et al., 2010). However, such scales have been primarily used in specific contexts, focusing on populations such as physicians and nurses (Huang and Chang, 2023). Few studies have assessed critical thinking attitudes in college students. Sosu (2013) developed the Critical Thinking Dispositions Scale, a 2-factor, 11-item tool, which measures two fundamental dispositions of CT: critical openness and reflective scepticism. Although research is limited, initial assessments suggest that the scale has good internal consistency and convergent validity (Yockey, 2016). However, Sosu’s (2013) sample involved college students and graduates enrolled in a program (specifically education), which could potentially influence the nature of the responses. Recently, Quinn et al. (2020) developed Student-Educator Negotiated Critical Thinking Dispositions Scale (SENCTDS), a tool composed of 21 items, measuring six dispositions: reflection, attention, open-mindedness, organization, perseverance, and intrinsic goal motivation. Despite the evaluation on a not very large sample, it has shown good validity and reliability. Stupple et al. (2017) developed the Critical Thinking Toolkit (CriTT): a measure of students’ attitudes and beliefs regarding critical thinking, based on three latent factors identified through a reduced set of 27 items. These factors were characterized as: confidence in critical thinking; valuing critical thinking; and misconceptions. Reliability analysis confirmed that the subscales were reliable. Additionally, convergent validity was demonstrated with measures of grade point average and argumentation ability. The CTAS (Critical Thinking Attitude Scale) initially developed by Akar and Kara (2020) to assess students’ critical thinking attitudes, consists of 20 items and includes seven dimensions (open-mindedness, problem-solving, fatalism, paranormal beliefs, dogmatism, conservatism, and discussion). Correlations between the subscales of the scale and the total score ranged from 0.43 to 0.67.

In summary, the CCTDI is a widely used tool, but doubts have been raised about its validity and reliability. Reduced partial versions, such as those by Hwang et al. (2010) and Wang et al. (2019), have shown increased reliability but have been mainly used in specific contexts such as the medical field. Other instruments, such as Sosu’s Critical Thinking Dispositions Scale (Sosu, 2013) and Quinn et al.’s SENCTDS (Quinn et al., 2020), focus on specific aspects of critical thinking, with promising but limited results. Alternatives for assessment include Stupple et al.'s (2017) CriTT and Akar and Kara (2020) CTAS scale. The process of developing a test suitable for the population of Italian university students requires formulating clear and understandable questions, reducing the test to eliminate duplications, and refining scale items to ensure accurate measurement of individual propensity toward critical thinking.

### Unique contribution of the current study

Existing measures of critical thinking attitude present notable limitations. The CCTDI, while widely used, is often criticized for its excessive length, which can lead to participant fatigue, reduced attention, and potential biases in responses (Bowling et al., 2022). Shortened versions of the CCTDI (Hwang et al., 2010; Wang et al., 2019) have improved reliability, yet their validation has been largely confined to medical and nursing students, thus limiting generalizability to broader academic contexts. Other instruments, such as Sosu’s (2013) Critical Thinking Dispositions Scale and Quinn et al.’s SENCTDS (Quinn et al., 2020), show promising psychometric properties, but they have been tested on relatively small or discipline-specific samples. Overall, these limitations highlight the need for instruments that are both psychometrically sound and broadly applicable across different academic domains.

The present study addresses this gap by providing the first Italian adaptation and validation of the Critical Thinking Attitude Scale (CTAS). Beyond mere language translation, this adaptation involved testing the factorial structure, refining items for cultural and linguistic appropriateness, and establishing the reliability and validity of the instrument in a large sample of Italian university students. This work therefore offers a standardized and contextually adapted measure of critical thinking attitude, suitable for educational research and practical applications in higher education.

### The present study

The aim of the current study was to develop a new measure of the attitude to critical thinking, drawing on previous theories and scales. Although there was a general conceptual model encompassing various aspects of disposition to critical thinking identified in previous literature (Hwang et al., 2010; Sosu, 2013; Akar and Kara, 2020; Quinn et al., 2020). The present study aimed to develop and standardize in Italian college students the construct of the attitude to critical thinking.

## Methods

This study follows a rigorous multi-phase psychometric validation process. Initially, a preliminary phase was conducted involving item development, expert review, and pilot testing. Subsequently, Study 1 employed exploratory factor analysis to identify the underlying factor structure and refine the scale with a smaller sample. Finally, Study 2 used confirmatory factor analysis with a larger sample to validate the factor structure, assess internal consistency reliability, and examine convergent and divergent validity of the instrument.

### Preliminary phase—item generation

In the preliminary phase of constructing the Critical Thinking Attitude Scale (CTAS), a systematic approach was adopted to generate elements, translate them and evaluate them with expert input, conduct pilot tests on the discrimination index, and refine the questionnaire. This phase provided the foundation for subsequent validation procedures aimed at ensuring the psychometric robustness of the CTAS. Based on validated instruments, well-established theoretical frameworks, and the construct definition, we developed an initial pool of 32 items intended to capture different facets of critical thinking attitude, including problem solving, systematicity, openness, truth-seeking, paranormal beliefs, and inquisitiveness (see Table 1 for item sources). The preliminary pool was then translated into Italian and culturally adapted.

**Table 1:** Critical thinking attitude scale (CTAS): item content and sources.

Items	Content	Source
I know how to think systematically.	Problem solving	Hwang et al. (2010)
I am able to think logically.	Problem solving	Hwang et al. (2010)
I draw conclusions through precise logical and methodological analyses.	Confidence in critical thinking—systematicity	Stupple et al. (2017)
I concentrate continuously when I have to tackle a problem.	Systematic analysis	Hwang et al. (2010)
People say that I make decisions meticulously.	Systematic analysis	Hwang et al. (2010)
I can connect new data with what I already know.	Confidence in critical thinking—systematicity	Stupple et al. (2017)
I can read between the lines and find contradictions among the various parts of the text.	Confidence in critical thinking—systematicity	Stupple et al. (2017)
I can relate the results of observation to existing theories of knowledge.	Confidence in critical thinking—systematicity	Stupple et al. (2017)
Others turn to me to solve their problems.	Systematic analysis	Hwang et al. (2010)
I am curious to get to the bottom of things.	Thinking outside the box—search for truth and openness	Hwang et al. (2010)
I always try to delve deeper and understand things thoroughly.	Thinking outside the box—search for truth and openness	Hwang et al. (2010)
When something does not convince me, I examine all possible alternatives.	Open-mindedness—search for truth and openness	Quinn et al. (2020)
I am convinced that we should learn everything we can; you never know when it might come in handy.	Thinking outside the box—search for truth and openness	Hwang et al. (2010)
I always try to understand the ideas of others.	Thinking outside the box—search for truth and openness	Hwang et al. (2010)
I expect to be able to face the challenges of life.	Attention-analyticity	Stupple et al. (2017) and Quinn et al. (2020)
Every evaluation we make must be based on criteria.	Attention-analyticity	Stupple et al. (2017) and Quinn et al. (2020)
I can assess the value of each piece of information in a problem.	Thinking within the box-analyticity	Hwang et al. (2010)
I make sure that each piece of information is reliable before putting them together in the problem.	Thinking within the box-analyticity	Hwang et al. (2010)
If I have to work on a problem, I can clear my mind of everything else.	Thinking within the box-attention-analyticity	Hwang et al. (2010), Quinn et al. (2020)
Some people have the gift of clairvoyance, meaning the ability to know the future.	Paranormal beliefs	Akar and Kara (2020)
Extraterrestrials have visited Earth in the past.	Paranormal beliefs	Akar and Kara (2020)
The positions of stars and planets can influence people’s lives.	Paranormal beliefs	Akar and Kara (2020)
I believe curses are effective.	Paranormal beliefs	Akar and Kara (2020)
The spirits of deceased individuals can return in certain places and situations.	Paranormal beliefs	Akar and Kara (2020)
I know how to use rigorous investigative methods to solve problems.	Critical Openness -Inquisitiveness	Sosu (2013)
It would be wonderful to study new things for a lifetime.	Inquisitiveness	Stupple et al. (2017) and Quinn et al. (2020)
I have a great desire to learn new things.	Critical openness-inquisitiveness	Sosu (2013)
I am tolerant in welcoming ideas that are different from mine.	Critical openness-inquisitiveness	Sosu (2013)
I always seek the most reliable sources when I need to understand a problem.	Critical openness -Inquisitiveness	Sosu (2013)
I am curious to investigate even the phenomena that science has not yet explained.	Critical openness-Inquisitiveness	Sosu (2013)
When I analyse information, I try to be objective and honest.	Critical openness-inquisitiveness	Sosu (2013)
Before making a decision, I try to do everything possible to gather all the information.	Inquisitiveness	Hwang et al. (2010), Stupple et al. (2017)

### Preliminary phase—translation and cultural adaptation

The items of Table 1 were translated and culturally adapted following best practices for cross-cultural test adaptation (Beaton et al., 2000). Two independent bilingual translators produced parallel translations of the 32 items into Italian. A third bilingual expert compared and reconciled the versions, producing a single preliminary draft. This draft was then back translated into English by an independent translator who was blinded to the original items. Discrepancies between the original and the back-translated versions were discussed and resolved by the research team, with the support of two domain experts, to ensure semantic and conceptual equivalence. Finally, a small group of Italian university students (*n* = 15) participated in cognitive interviews to verify clarity, readability, and cultural appropriateness. Minor wording adjustments were made accordingly, and the final Italian version of the 32 items was retained for expert review and pilot testing.

### Preliminary phase—expert review and pilot testing

The resulting 32 items were reviewed by an independent panel of three experts in psychometrics, educational psychology, and critical thinking research, selected based on their publication record and expertise in test construction.

Each expert independently evaluated the items on relevance, clarity, and representativeness using a 5-point Likert scale. Inter-rater agreement was assessed for each item using Cohen’s *κ*, with an average κ of 0.78, indicating substantial agreement across reviewers. Items that received a mean score lower than 4 from at least two experts were revised or eliminated.

A pilot test of the questionnaire was then conducted with 72 participants to identify potential issues and assess each item’s ability to discriminate between high- and low-scoring individuals. The discrimination index (D; Chiorri, 2011) was applied, with items below 0.20 removed. This cutoff was chosen in line with established recommendations, to ensure that retained items provided sufficient differentiation between respondents. Items were removed only if they showed both low discrimination and weak theoretical relevance. This process led to the elimination of six items, leaving 26 items (see Table 2). The low discrimination indices for the removed items indicate that participants responded similarly regardless of their overall attitude toward critical thinking. For example, items assessing paranormal beliefs were uniformly endorsed at low levels, and the item “I expect to be able to face life’s challenges” received uniformly high responses, reflecting personal willpower rather than critical thinking attitude.

**Table 2:** Discrimination indices (DI) for each item.

Items	DI
1. I know how to think systematically.	0.68
2. I am able to think logically.	0.67
3. I draw conclusions through precise logical and methodological analyses.	0.55
4. I concentrate continuously when I have to tackle a problem.	0.57
5. People say that I make decisions meticulously.	0.49
6. I can connect new data with what I already know.	0.60
7. I can read between the lines and find contradictions among the various parts of the text.	0.64
8. I can relate the results of observation to existing theories of knowledge.	0.63
9. Others turn to me to solve their problems.	0.55
10. I am curious to get to the bottom of things.	0.65
11. I always try to delve deeper and understand things thoroughly.	0.69
12. When something does not convince me, I examine all possible alternatives.	0.71
13. I am convinced that we should learn everything we can; you never know when it might come in handy.	0.57
14. I always try to understand the ideas of others.	0.54
15. I expect to be able to face the challenges of life.	0.11
16. Every evaluation we make must be based on criteria.	0.69
17. I can assess the value of each piece of information in a problem.	0.62
18. I make sure that each piece of information is reliable before putting them together in the problem.	0.61
19. If I have to work on a problem, I can clear my mind of everything else.	0.55
20. Some people have the gift of clairvoyance, meaning the ability to know the future.	0.08
21. Extraterrestrials have visited Earth in the past.	0.11
22. The positions of stars and planets can influence people’s lives.	0.09
23. I believe curses are effective.	0.09
24. The spirits of deceased individuals can return in certain places and situations.	0.11
25. I know how to use rigorous investigative methods to solve problems.	0.61
26. It would be wonderful to study new things for a lifetime.	0.63
27. I have a great desire to learn new things.	0.56
28. I am tolerant in welcoming ideas that are different from mine.	0.59
29. I always seek the most reliable sources when I need to understand a problem.	0.70
30. I am curious to investigate even the phenomena that science has not yet explained.	0.72
31. When I analyse information, I try to be objective and honest.	0.51
32. Before making a decision, I try to do everything possible to gather all the information.	0.63

Although the resulting factors contained unequal numbers of items (ranging from 4 to 9), this structure was retained to preserve content validity and breadth of the construct. The factorial structure and internal consistency of each dimension were subsequently evaluated in Study 1.

### Study 1

The primary objective of this study was to investigate and validate the twenty-six items of Critical Thinking Attitude Scale obtained from the preliminary phase within the Italian student. This validation also aims to contribute to the understanding of the critical thinking attitude in Italian student.

#### Participants

All participants willingly agreed to take part in the study and completed a written informed consent. After the signed consent they fill out the CTAS scale. The sample consisted of 211 individuals, including 114 females and 97 males, with ages ranging from 18 to 30 years (M = 24.83, SD = 5.84). All participants were of Italian nationality, with 53% from Southern Italy, 24% from the North, and the remaining 23% from the Central regions. The selected students come from a wide range of academic departments, including economics, law, psychology, sciences, and engineering. This selection aims to ensure diverse representation, considering the various disciplines present in the academic environment. The recruitment process was conducted through various channels, such as classroom announcements, informative emails, and presentations during lectures. The goal was to include students at various stages of their academic journey, to obtain a broad range of perspectives and educational backgrounds. Data collection took place between January 2024 and March 2024. Participants completed the questionnaire online through a link provided via email. The survey was completed anonymously and individually, using the Google Forms platform. Regarding education level, 38% had completed high school, and 62% had at least a bachelor’s degree.

#### Statistical analysis

Exploratory factor analysis using IBM SPSS Statistics 24 was conducted to identify underlying dimensions and create internally consistent scales. Principal axis factoring (PAF) with oblique rotation was employed. Three criteria were used to extract the factors: parallel analysis of Monte Carlo simulations, an eigenvalue greater than 1 (Kaiser’s criterion), and visual inspection of the tables for significant contributions to the accounted variance. The internal consistency of the scales was evaluated using Cronbach’s alphas, with values of at least 0.7 considered acceptable and values of 0.8 or higher considered good. The alpha level was set at 0.05.

### Study 2

The primary objective of Study 2 was to establish the construct validity of the CTAS through a comprehensive examination of its factorial structure and concurrent validity. The study aimed to validate the previously identified four-factor structure, encompassing “Systematicity,” “Search for Truth and Openness,” “Analyticity,” and “Inquisitiveness,” employing a larger sample of adults than in Study 1. To explore convergent validity, two distinct measures were employed. Firstly, the Critical Reasoning Assessment (CRA) (Anghel et al., 2021; Fabio et al., 2025a, 2025b) served as a performance measure, assessing participants’ critical thinking abilities. Secondly, the Italian Big Five Inventory 10 personality test (BFI-10; Guido et al., 2015) was utilized, with a specific focus on the conscientiousness and openness components. These personality traits would exhibit significant correlations with individuals’ attitude towards critical thinking. This expectation was grounded in the theoretical assumption that conscientious individuals are likely to engage in systematic and careful thinking, while open individuals are predisposed to truth-seeking and openness to diverse perspectives. To assess divergent validity, the Dysfunctional Beliefs Questionnaire (DBQ) (Beck and Beck, 1991; Fabio et al., 2021) was employed. The rationale behind this choice lies in the expectation that scores on the Irrational Thoughts Scale would exhibit an inverse correlation with individuals’ critical thinking attitude. This anticipation is theoretically grounded in the notion that an attitude towards critical thinking involves a propensity to approach problems and decision-making with rational and evidence-based reasoning, thus indicating an inherent opposition to irrational thought patterns.

#### Participants

As in Study 1, participants were recruited through various channels, such as classroom announcements, informative emails, and presentations during lectures. They then completed the online questionnaire via a link sent by email, which directed them to Google Forms. Data collection took place between September 2024 and December 2024. All participants willingly agreed to take part in the study and completed written informed consent. The sample comprised 577 students, with 270 females and 307 males, ranging in age from 18 to 40 years (M = 28.75, SD = 6.45). All participants were of Italian nationality, with 48% from the Southern region, 37% from the Northern region, and the remaining 15% from the Central region. Regarding educational attainment, 27% had completed high school, and 73% had at least a bachelor’s degree.

#### Measures

The Critical Thinking Attitude Scale (CTAS) was used to examine results. The final 26-item CTAS from Study 1 was utilized to measure the four factors “systematicity” (nine items), “Search for Truth and Openness” (six items), “Analyticity” (four items) and “Inquisitiveness” (seven items). Participants responded on a Likert scale ranging from 1 (strongly disagree) to 5 (strongly agree), where a lower score corresponds to a lower critical thinking attitude, while a higher score indicates a higher critical thinking attitude, reflecting greater systematicity, search for truth and openness, analyticity, and inquisitiveness.

The Critical Reasoning Assessment (CRA) (Anghel et al., 2021; Fabio et al., 2025a, 2025b) is grounded in the well-established Reflective Judgment Model by King and Kitchener (2004) to objectively assess critical thinking skills through specific tasks. The structure of the CRA employs three specially designed dilemmas to stimulate critical thinking on key themes: genetics vs. choice, fairness, and compassion. Participants undergoing the CRA respond to targeted questions related to each dilemma, exploring their decision-making process, and delving into the foundations of their viewpoint. The CRA assessment comprises five dimensions: cognitive complexity, reasoning style, openness, nature of knowledge, and nature of justification. Each dimension is evaluated on a scale from 1 to 7 (refer to the Supplementary material), providing a detailed analysis of participants’ critical thinking. The tool demonstrates reliability, with a Cronbach’s alpha coefficient of *α* = 0.87. In the study, participants’ responses, assessed by two experts, exhibit a high level of agreement with a Cohen’s Kappa concordance coefficient of 0.96.

The Dysfunctional Beliefs Questionnaire (DBQ) (Beck and Beck, 1991), validated and adapted into Italian by Fabio et al. (2021), aims to identify a broad set of dysfunctional beliefs in individuals considered psychologically healthy. The 36 questionnaire items reflect common dysfunctional beliefs associated with everyday life events and are structured into four subscales measuring self-criticism and devaluation, catastrophizing, absolute duty, and intolerance of frustration. Participants express their responses using a 5-point Likert scale, where higher scores indicate a greater level of dysfunctional beliefs. Internal consistency analysis has confirmed that the DBQ exhibits high overall reliability, standing at 90%.

The Italian BFI-10 (Guido et al., 2015) represents an abbreviated version of the Big Five Inventory (BFI) developed by Rammstedt (2007). The scale’s structure involves a preliminary statement, namely “I see myself as someone who…” Subsequently, participants are presented with a total of ten statements, two to assess each of the five personality dimensions: Agreeableness, Conscientiousness, Emotional Stability, Extraversion, and Openness. For each statement, participants are asked to respond on a five-point Likert scale, ranging from 1 (strongly disagree) to 5 (strongly agree). Spearman–Brown coefficients, employed to assess the internal consistency of the scale, demonstrated a value equal to or exceeding 0.78.

#### Statistical analysis

Confirmatory factor analyses (CFA) were conducted using JASP (version X.X) to validate the four-factor structure of the CTAS identified in Study 1. The analyses employed maximum likelihood (ML) estimation, which is appropriate for continuous, approximately normally distributed data. Model fit was evaluated using multiple indices, including the chi-square to degrees of freedom ratio (χ^2^/df), Comparative Fit Index (CFI), Goodness of Fit Index (GFI), and Root Mean Square Error of Approximation (RMSEA). To further assess the scale’s validity, correlations with other constructs were examined: Pearson correlations were calculated between the CTAS and CRA, DBQ, and BFI-10, while Spearman rank correlations were used to explore relationships between the CTAS and performance-based critical thinking measures, dysfunctional beliefs, and personality traits. Internal consistency of the CTAS was assessed using Cronbach’s alpha.

## Results

### Study 1

The Kaiser-Meyer-Olkin (KMO) measure of sampling adequacy was 0.91, indicating that the correlation matrix was suitable for factor analysis. Table 3 displays the factor loadings for all items, showing high loadings on four factors. The first factor accounted for 28.1% of the variance, the second factor explained 13.5%, the third factor explained 13.4%, and the fourth explained 8.2% of the variance, with a cumulative explained variance of 63.2%.

**Table 3:** Factor loadings based on the EFA.

Item	RC1	RC2	RC3	RC4
1. I know how to think systematically.	0.736			
2. I’m able to think logically.	0.712			
3. I draw conclusions through precise logical and methodological analyses.	0.651			
4. I concentrate continuously when I have to tackle a problem.	0.641			
5. People say that I make decisions meticulously.	0.628			
6. I can connect new data with what I already know.	0.603			
7. I can read between the lines and find contradictions among the various parts of the text.	0.561			
8. I can relate the results of observation to existing theories of knowledge.	0.561			
9. Others turn to me to solve their problems.	0.455			
10. I am curious to get to the bottom of things.		0.723		
11. I always try to delve deeper and understand things thoroughly.		0.711		
12. When something does not convince me, I examine all possible alternatives.		0.595		
13. I am convinced that we should learn everything we can; you never know when it might come in handy.		0.579		
14. I always try to understand the ideas of others.		0.559		
16. Every evaluation we make must be based on criteria.		0.490		
17. I can assess the value of each piece of information in a problem.			0.771	
18. I make sure that each piece of information is reliable before putting them together in the problem.			0.733	
19. If I have to work on a problem, I can clear my mind of everything else.			0.543	
25. I know how to use rigorous investigative methods to solve problems.			0.412	
26. It would be wonderful to study new things for a lifetime.				0.652
27. I have a great desire to learn new things.				0.624
28. I am tolerant in welcoming ideas that are different from mine.				0.523
29. I always seek the most reliable sources when I need to understand a problem.				0.516
30. I am curious to investigate even the phenomena that science has not yet explained.				0.503
31. When I analyse information, I try to be objective and honest.				0.493
32. Before making a decision, I try to do everything possible to gather all the information.				0.488

Although item numbers in this table range up to 32, the EFA was performed on the 26-item version of the CTAS. Six items from the initial 32-item pool were discarded during pilot testing due to low theoretical relevance and poor discrimination and therefore were not included in the factor analysis. The numbering in this table corresponds to the original preliminary item pool.

The first factor, termed “Systematicity,” is characterized by the strong loading of nine items and reflects participants’ inclination to approach problem-solving in a disciplined and organized manner. Key items captured within this factor include statements such as “I am capable of thinking systematically” and “I draw conclusions through precise logical and methodological analyses.” The second factor, denoted as “Search for Truth and Openness,” encompasses six items and underscores participants’ rational reflection grounded in evidence. It involves avoiding biases, embracing diverse opinions, and expressing oneself courageously. Examples of items within this category include “I am curious to delve into issues” and “I am satisfied with my ability to understand others’ ideas.” The third factor, titled “Analyticity,” comprises four items and focuses on the competence to meticulously examine and evaluate information for a comprehensive understanding and accurate judgment of situations. Sample items in this category might encompass statements such as “I can use investigative methods to solve problems” and “I ensure that every piece of information is reliable before integrating it into problem resolution.” The fourth factor is identified as “Inquisitiveness,” featuring seven items that pertain to the eagerness to acquire knowledge. Examples of items within this factor include statements like “It would be wonderful to study new things throughout life” and “I have a strong desire to learn new things”.

Scores for each subscale were created by summing the corresponding items. Table 4 presents the average scores for each subscale: Systematicity (M = 33.37, SD = 7.81), Search for Truth and Openness (M = 25.51, SD = 3.73), Analyticity (M = 15.90, SD = 3.23), and Inquisitiveness (M = 26.35, SD = 34.26). The mean and standard deviation of the full CTAS were 101.15 and 13.13. No gender differences emerged for either the total scale or the subscales. The Cronbach’s alphas for the four factors were very high: 0.94, 0.83, 0.92, 0.78 respectively, and 0.89 for the full scale. All the subscales showed significant correlations with the CTAS.

**Table 4:** Descriptive statistics and correlations for CTAS.

Factor	M	SD	α	1	2	3	4	5
1. CTAS	101.15	13.13	0.89	–				
2. Systematicity	33.37	7.81	0.94	0.849**	–			
3. Search for truth and openness	25.51	3.73	0.83	0.690**	0.438**	–		
4. Analyticity	15.90	3.23	0.92	0.699**	0.501**	0.445**	–	
5. Inquisitiveness	26.35	4.26	0.78	0.389**	0.020	0.109**	0.088	–

CTAS = Critical Thinking Attitude Scale. Systematicity (9 items; range = 9–45), Search for Truth and Openness (6 items; range = 6–30), Analyticity (4 items; range = 4–20), Inquisitiveness (7 items; range = 7–35), and CTAS Total (26 items; range = 26–130). Responses were given on a 5-point Likert scale (1 = strongly disagree to 5 = strongly agree), with higher scores indicating stronger critical thinking attitudes. α = Cronbach’s alpha. *p* < 0.01**.

### Study 2

#### Confirmatory factor analysis

Confirmatory factor analyses (CFA) were conducted using JASP (version X.X) to validate the four-factor structure of the CTAS identified in Study 1. Maximum likelihood (ML) estimation was employed, suitable for continuous, approximately normally distributed data. Model fit was evaluated using multiple indices: chi-square to degrees of freedom ratio (χ^2^/df), Comparative Fit Index (CFI), Goodness of Fit Index (GFI), and Root Mean Square Error of Approximation (RMSEA). The CFA confirmed the four-factor structure corresponding to the subscales identified in Study 1:

Systematicity (9 items: 1–9), reflecting disciplined and organized problem-solving;
Search for Truth and Openness (6 items: 10–14,16), capturing curiosity, openness, and evidence-based reflection;
Analyticity (4 items: 17–19, 25), measuring careful evaluation and verification of information;
Inquisitiveness (7 items: 26–32), representing eagerness to acquire knowledge.

**Figure 1:**
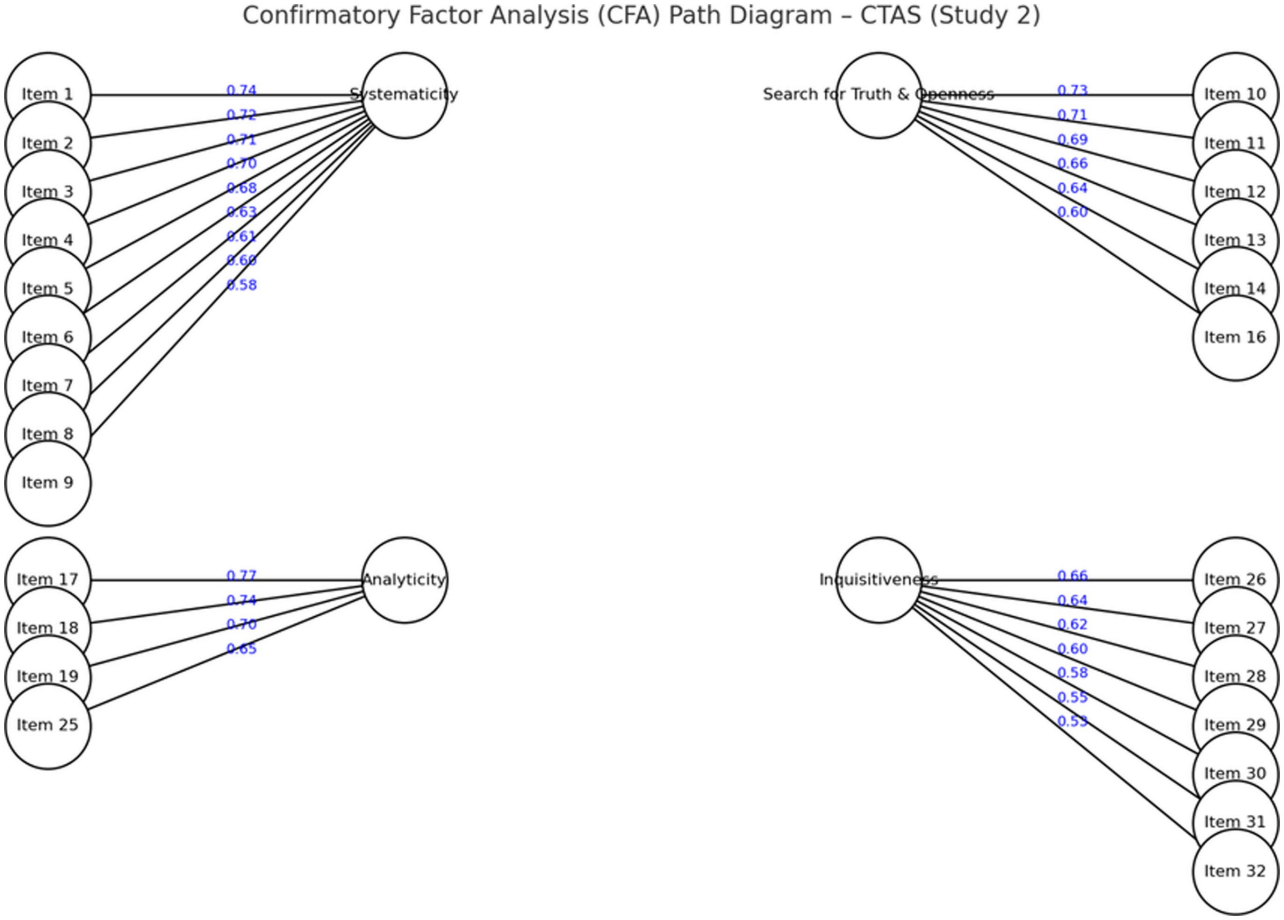
Final CFA model of the CTAS with freed error covariances.

All factor loadings were above 0.53, indicating strong relationships between items and their respective factors (see Figure 1).

To improve model fit, four covariances between error terms were freed. These modifications were theoretically justified because the paired items shared highly similar content within the same latent factor:

Items 1↔3 (systematicity: logical and methodological precision)
Items 2↔4 (systematicity: sustained attention and careful reasoning)
Items 10↔11 (search for truth and openness: curiosity and depth of inquiry)
Items 26↔27 (inquisitiveness: eagerness to acquire knowledge)

After these adjustments, the model demonstrated excellent fit: χ^2^(576) = 14.81, *p* < 0.07, CFI = 0.965, RMSEA = 0.067 (90% CI [0.049, 0.076]). An alternative bifactor model, including a general critical thinking attitude factor alongside the four specific factors, yielded comparable fit indices (χ^2^/df = 1.48, CFI = 0.963, RMSEA = 0.068, 90% CI [0.050, 0.077]), confirming the stability of the four-factor structure.

Figure 1 shows the final CFA path diagram, including the four latent factors, item loadings, and the four freed covariances between error terms.

#### Reliability

A Cronbach’s alpha value of 0.93 indicated that the twenty-six-item CTAS had satisfactory to excellent internal consistency. Test–retest reliability was calculated on a subsample of 111 participants four weeks after the initial administration, yielding a coefficient of 0.97, indicating a high level of stability. Cronbach’s *α* coefficients for the four subscales were also satisfactory: systematicity (α = 0.91), Search for Truth and Openness (α = 0.88), Analyticity (α = 0.86), and Inquisitiveness (α = 0.89). McDonald’s *ω* ranged from 0.88 to 0.92 across the four subscales, and composite reliability (CR) values ranged from 0.87 to 0.93, both indicating strong reliability (Cronbach, 1951; McDonald, 1999; Tavakol and Dennick, 2011).

To further evaluate internal consistency, additional indices were computed. McDonald’s ω ranged from 0.88 to 0.92 across the four subscales, and composite reliability (CR) values ranged from 0.87 to 0.93, both indicating strong reliability. The average variance extracted (AVE) ranged from 0.54 to 0.63, suggesting that each factor captured a substantial proportion of variance relative to measurement error. Item–total correlations were all above 0.40, and no item deletion substantially improved reliability coefficients, suggesting that item redundancy was not problematic despite the high alpha values.

#### Convergent and criterion validity

To assess the convergent analysis of the CTAS, Pearson’s coefficient correlations between the CTAS, CRA, DBQ, and BFI-10 were performed. As shown in Table 5, the CTAS was significantly and positively correlated with CRA and BIG-10 while it was negatively correlated with DBQ. This indicates that higher scores on the CTAS (indicating higher levels of CT attitude) were associated with higher scores on the subscales of CRA and BFI-10.

**Table 5:** Correlations between CTAS, and CRA, DBQ, and BIG-5.

Variable	CTAS	Systematicity	Search for Truth and Openness	Analyticity	Inquisitiveness
*CRA*	0.389**	0.414**	0.338**	0.236**	0.396**
Cognitive complexity	0.283*	0.299*	0.257*	0.279*	0.266*
Reasoning style	0.301**	0.443**	0.408**	0.296**	0.344**
Openness	0.413**	0.409**	0.424**	0.306**	0.338**
Nature of knowledge	0.212	0.313*	0.191	0.121	0.173
Nature of justification	0.122	0.338*	0.215	0.182	0.209
*DBQ*	−0.359**	−0.442**	−0.309**	−0.382**	−0.354**
Self-criticism and devaluation	−0.328**	−0.368**	−0.324**	−0.306**	−0.331**
Catastrophizing	−0.381**	−0.372**	−0.358**	−0.314**	−0.311**
Absolute duty	−0.209**	−0.299**	−0.302**	−0.342**	−0.309**
Intolerance of frustration	−0.312**	−0.356**	−0.299**	−0.344**	−0.322**
*BIG-5*					
Openness	0.605**	0.459**	0.350**	−0.24	0.348**
Conscientiousness	0.480**	0.525**	0.389**	0.017	0.386**
Extraversion	−0.017	−0.096	0.108	−0.016	0.101
Agreeableness	−0.141	−0.023	0.017	−0.136	0.014
Neuroticism	−0.146	−0.091	0.009	0.046	0.005

* = *p* < 0.05; ** = *p* < 0.01.

At the subscale level, systematicity and analyticity were most strongly associated with the CRA dimension of cognitive complexity (r = 0.41 and r = 0.38, respectively, *p* < 0.001). Search for Truth and Openness correlated positively with CRA openness (r = 0.36, *p* < 0.001) and with Big Five openness (r = 0.32, *p* < 0.001). Inquisitiveness was positively related to Extraversion (r = 0.29, *p* < 0.001) and negatively associated with dysfunctional beliefs, particularly catastrophizing (r = −0.34, *p* < 0.001). These results provide evidence of both convergent and criterion validity, showing that higher CTAS scores align with theoretically related constructs of critical reasoning, adaptive personality traits, and lower dysfunctional beliefs.

## Discussion

The present study aimed to develop and validate the Critical Thinking Attitude Scale (CTAS) for Italian university students, providing a culturally and linguistically adapted instrument to systematically assess attitudes toward critical thinking. The results support the scale’s validity and reliability in this population.

In Study 1, exploratory factor analysis identified a clear four-factor structure: *Systematicity*, *Search for Truth and Openness*, *Analyticity*, and *Inquisitiveness*. This structure was confirmed in Study 2 through confirmatory factor analysis on a larger sample, showing excellent fit indices (χ^2^/df = 14.81; CFI = 0.965; RMSEA = 0.067), with item factor loadings ranging from 0.53 to 0.87, confirming the robustness of the factors. Internal consistency was high across all dimensions (Systematicity *α* = 0.91, Search for Truth and Openness α = 0.88, Analyticity α = 0.86, Inquisitiveness α = 0.89; total α = 0.93), and test–retest reliability indicated excellent temporal stability (r = 0.97).

Correlations with other instruments further support both the convergent and divergent validity of the CTAS. Positive correlations with the Critical Reasoning Assessment (CRA; Anghel et al., 2021; Fabio et al., 2025a, 2025b) suggest that students with stronger critical thinking attitudes perform better on objective reasoning tasks. Similarly, significant positive associations with Conscientiousness (r = 0.35, *p* < 0.001) and Openness (r = 0.32, *p* < 0.001) from the BFI-10 indicate that students who are more conscientious and open-minded are more likely to exhibit a developed critical thinking attitude. In contrast, the negative correlation with the Dysfunctional Beliefs Questionnaire (DBQ; r = −0.34, *p* < 0.001) confirms that the CTAS assesses a construct distinct from dysfunctional beliefs. These findings align with the dual-process theory (Stanovich, 2011), suggesting that higher conscientiousness and openness relate to System 2 processes, characterized by deliberate and reflective reasoning, whereas dysfunctional beliefs are more closely linked to automatic, heuristic-driven System 1 processes.

The results also align with, and extend, previous research. For example, Rosnawati et al. (2015) reported factor loadings between 0.66 and 0.76 for comparable dimensions, while Syahfitri et al. (2019) found loadings ranging from 0.51 to 0.94, supporting the multidimensional nature of critical thinking. However, these prior studies were often restricted to specific disciplines or small samples, without providing an integrated assessment of critical thinking attitude. The Italian CTAS addresses this by capturing multiple facets of critical thinking attitude across students from diverse academic programs, facilitating broader comparisons and supporting targeted educational interventions.

Context and educational experience remain important determinants of critical thinking attitudes. Selda (2015) observed differences among teacher-training students based on disciplinary background, while Abrami et al. (2015) demonstrated that authentic problem-solving and mentoring improve analytical and reflective abilities. Huber and Kuncel (2016) similarly highlighted that both critical thinking skills and attitudes can be strengthened through structured interventions. The Italian CTAS captures these variations, showing that students with higher *Systematicity* and *Analyticity* engage in more structured, reflective reasoning, whereas those with higher *Search for Truth and Openness* and *Inquisitiveness* demonstrate greater curiosity and openness to new information and perspectives.

These results are consistent with the Reflective Judgment Model by King and Kitchener (2004), which proposes that critical thinking develops from reliance on direct sources toward greater awareness of uncertainty and eventually to informed evaluation of evidence. This underscores the importance of educational strategies that foster not only cognitive skills but also attitudes toward critical thinking, promoting reflective, analytical, and adaptive reasoning.

In conclusion, the findings highlight the relevance of reliable, multidimensional assessment tools like the CTAS for measuring critical thinking attitudes. Such tools are essential for improving educational quality and preparing students to navigate complex real-world challenges with a reflective and evidence-based mindset.

### Study limitations

It is important to acknowledge some limitations of the present research. Despite efforts to involve students from different types of faculties, there may be a lack of complete representation of the Italian student population. This could influence the generalizability of the results. Furthermore, the specific environmental factors of the Italian educational context should not be overlooked, as they may influence students’ responses to the instrument. These factors may not have been fully considered or controlled in the study, potentially limiting our understanding of students’ critical thinking attitudes.

Another consideration concerns the interpretation of convergent and divergent validity. While correlations with the Big Five Inventory (BFI-10) and Dysfunctional Beliefs Questionnaire (DBQ) rely on self-report data, which may introduce common-method bias, it is important to highlight that the Critical Reasoning Assessment (CRA) is a performance-based measure evaluated by trained experts rather than a self-report instrument. Thus, the correlation with the CRA provides an objective indicator of critical thinking ability. Nevertheless, caution is warranted when interpreting correlations with self-report measures, and future studies should incorporate additional performance-based or behavioral assessments to further validate the CTAS and reduce potential biases associated with shared method variance.

Moreover, it should be noted that we did not examine measurement invariance across relevant subgroups such as gender or disciplinary field. Establishing invariance would allow stronger conclusions about the comparability of CTAS scores across different populations. The lack of such analyses represents a limitation, and future research should test the factorial stability of the instrument across subgroups to ensure that the scale operates equivalently in diverse student samples.

Finally, it is important to note that the present validation was conducted exclusively within the Italian cultural and educational context. Although critical thinking attitudes are theorized as universal dispositions, their expression and measurement may vary across cultural and disciplinary boundaries. Thus, the claims about applicability to all disciplines and broader educational contexts should be considered with caution. Future research should test the cross-cultural validity and measurement invariance of the CTAS, as well as its relevance across different academic disciplines, to ensure that the scale captures critical thinking attitudes in a comparable and culturally sensitive manner.

### Practical implications and future research

The study provides a valid and reliable tool for assessing critical thinking dispositions among Italian university students. While the CTAS has shown strong psychometric properties in this context, its applicability beyond the Italian higher education system cannot be assumed. Therefore, its use should be considered context-specific until additional evidence of generalizability becomes available. Educators in Italian universities may employ the CTAS to identify strengths and areas for development in students’ critical thinking, thereby supporting the design of targeted educational interventions.

Future research could further explore the application of the CTAS in specific contexts, such as academic settings. Critical thinking plays a crucial role in all academic disciplines, enabling students to critically analyze concepts, interpret information, and formulate valid arguments. The ability to critically evaluate information contributes to deeper and more meaningful learning. Challenges within the academic realm often require creative and well-considered solutions. Critical thinking enables students to identify and effectively solve problems, promoting a deeper understanding of the concepts and theories studied. The academic environment necessitates continuous learning and assimilation of new knowledge. Critical thinking fosters an open approach to learning, encouraging students to explore new perspectives and tackle challenges with confidence and competence. Finally, longitudinal studies could provide insights into the stability of students’ critical thinking attitudes over time.

## Conclusion

In summary, Critical Thinking Attitude Scale (CTAS) has proven to be a valid and reliable instrument for assessing critical thinking attitudes in the Italian college student. The results provide a solid foundation for further research and practical applications in educational settings. Advancements in the analysis and development of critical thinking positively contribute to individuals’ preparation to tackle the complex challenges of contemporary society.

## Data Availability

The raw data supporting the conclusions of this article will be made available by the authors, without undue reservation.

## References

[ref1] Abrami P. C. Bernard R. M. Borokhovski E. Waddington D. I. Wade C. A. Persson T. (2015). Strategies for teaching students to think critically: a meta-analysis. Rev. Educ. Res. 85, 275–314. doi: 10.3102/00346543145510

[ref2] Akar C. Kara M. (2020). Critical thinking attitude and some other variables in predicting students' democratic attitudes. Int. J. Contemp. Educ. Res. 7, 226–245. doi: 10.33200/ijcer.686662

[ref3] Anghel E. Braun H. I. Friedman A. A. Baez-Cruz M. (2021). College students' critical thinking: assessment and interpretation. J. High. Educ. Theory Pract. 21, 36–53. doi: 10.33423/jhetp.v21i10.4624

[ref4] Anum O. A. Olumuyiwa B. M. (2024). Perception of economics education and social studies education students’ on the impact of emotional intelligence and critical thinking in their course area. Afr. J. Educ. Found. 5, 42–55.

[ref5] Azzaakiyyah H. K. Wanof M. I. Suherlan S. Fitri W. S. (2023). Business philosophy education and improving critical thinking skills of business students. J. Contemp. Adm. Manag. 1, 1–4. doi: 10.61100/adman.v1i1.1

[ref6] Baddane K. Ennam A. (2024). Measuring pedagogical transformation: a quantitative analysis of critical thinking integration in literary criticism for heightened student engagement and learning outcomes. Int. J. Linguist. Lit. Transl. 7, 39–50. doi: 10.32996/ijllt.2024.7.1.4

[ref7] Beaton D. E. Bombardier C. Guillemin F. Ferraz M. B. (2000). Guidelines for the process of cross-cultural adaptation of self-report measures. Spine 25, 3186–3191. doi: 10.1097/00007632-200012150-00014, PMID: 11124735

[ref8] Beck A. T. Beck J. S. (1991). The personality belief questionnaire. Unpublished assessment instrument. Bala Cynwyd, PA: the Beck Institute for cognitive therapy and research. Accessed September 2025. Available online at: https://beckinstitute.org/wp-content/uploads/2021/06/PBQ-Full-Documents-1.pdf

[ref9] Bowling N. A. Gibson A. M. DeSimone J. A. (2022). Stop with the questions already! Does data quality suffer for scales positioned near the end of a lengthy questionnaire? J. Bus. Psychol. 37, 1099–1116. doi: 10.1007/s10869-021-09787-8

[ref10] Bravo M. J. Galiana L. Rodrigo M. F. Navarro-Pérez J. J. Oliver A. (2020). An adaptation of the critical thinking disposition scale in Spanish youth. Think. Skills Creat. 38:100748. doi: 10.1016/j.tsc.2020.100748

[ref001] Chiorri C. (2011). Teoria e tecnica psicometrica. McGraw-Hill.

[ref11] Cossu R. Awidi I. Nagy J. (2024). Critical thinking activities in fluid mechanics–a case study for enhanced student learning and performance. Educ. Chem. Eng. 46, 35–42. doi: 10.1016/j.ece.2023.10.004

[ref12] Cronbach L. J. (1951). Coefficient alpha and the internal structure of tests. Psychometrika 16, 297–334. doi: 10.1007/BF02310555

[ref13] Cui L. Zhu Y. Qu J. Tie L. Wang Z. Qu B. (2021). Psychometric properties of the critical thinking disposition assessment test amongst medical students in China: a cross-sectional study. BMC Med. Educ. 21, 1–8. doi: 10.1186/s12909-020-02437-2 33407421 PMC7786903

[ref14] Evans J. S. B. Stanovich K. E. (2013). Dual-process theories of higher cognition: advancing the debate. Perspect. Psychol. Sci. 8, 223–241. doi: 10.1177/1745691612460685, PMID: 26172965

[ref15] Fabio R. A. Caprì T. Buzzai C. Campana R. (2021). Construction and validation of an Italian dysfunctional beliefs’ questionnaire. Curr. Psychol. 40, 618–628. doi: 10.1007/s12144-018-9958-8

[ref16] Fabio R. A. Plebe A. Ascone C. Suriano R. (2025a). Psychometric properties and validation of the critical reasoning assessment. Pers. Individ. Differ. 246:113344. doi: 10.1016/j.paid.2025.113344

[ref17] Fabio R. A. Plebe A. Suriano R. (2025b). AI-based chatbot interactions and critical thinking skills: an exploratory study. Curr. Psychol. 44, 8082–8095. doi: 10.1007/s12144-024-06795-8

[ref18] Fabio R. A. Suriano R. (2021). The influence of media exposure on anxiety and working memory during lockdown period in Italy. Int. J. Environ. Res. Public Health 18, 237–260. doi: 10.3390/ijerph18179279, PMID: 34501866 PMC8430792

[ref19] Fabio R. A. Suriano R. (2023). The influence of smartphone use on tweens’ capacity for complex critical thinking. Children 10:698. doi: 10.3390/children10040698, PMID: 37189947 PMC10136902

[ref20] Fabio R. A. Suriano R. (2025). Thinking with humility: investigating the role of intellectual humility in critical reasoning performance. Pers. Individ. Differ. 244:113251. doi: 10.1016/j.paid.2025.113251

[ref21] Facione N. C. Facione P. A. Sanchez C. A. (1994). Critical thinking disposition as a measure of competent clinical judgment: the development of the California critical thinking disposition inventory. J. Nurs. Educ. 33, 345–350. doi: 10.3928/0148-4834-19941001-05, PMID: 7799093

[ref23] Guido G. Peluso A. M. Capestro M. Miglietta M. (2015). An italian version of the 10-item big five inventory: an application to hedonic and utilitarian shopping values. Pers. Individ. Differ. 76, 135–140. doi: 10.1016/j.paid.2014.11.053

[ref24] Huang X. Chang Y. C. (2023). Critical thinking instruction incorporated in cross-cultural communication course design: a needs analysis report based on voices of Chinese international college undergraduates. J. Educ. Learn. 12, 40–51. doi: 10.5539/jel.v12n1p40

[ref25] Huber C. R. Kuncel N. R. (2016). Does college teach critical thinking? A meta-analysis. Rev. Educ. Res. 86, 431–468. doi: 10.3102/0034654315605917

[ref26] Hwang S. Y. Yen M. Lee B. O. Huang M. C. Tseng H. F. (2010). A critical thinking disposition scale for nurses: short form. J. Clin. Nurs. 19, 3171–3176. doi: 10.1111/j.1365-2702.2010.03343.x, PMID: 21040020

[ref27] Hwang G. J. Zou D. Wu Y. X. (2023). Learning by storytelling and critiquing: a peer assessment-enhanced digital storytelling approach to promoting young students’ information literacy, self-efficacy, and critical thinking awareness. Educ. Technol. Res. Dev., 1–25. doi: 10.1007/s11423-022-10184-y

[ref28] İlaslan E. Adıbelli D. Teskereci G. Cura Ş. Ü. (2023). Development of nursing students' critical thinking and clinical decision-making skills. Teach. Learn. Nurs. 18, 152–159. doi: 10.1016/j.teln.2022.07.004

[ref29] Kaczkó É. Ostendorf A. (2023). Critical thinking in the community of inquiry framework: an analysis of the theoretical model and cognitive presence coding schemes. Comput. Educ. 193:104662. doi: 10.1016/j.compedu.2022.104662

[ref30] Karaer G. Hand B. French B. F. (2024). Examining the impact of science writing heuristic (SWH) approach on development of critical thinking, science and language skills of students with and without disabilities. Think. Skills Creat. 51:101443. doi: 10.1016/j.tsc.2023.101443

[ref31] King P. M. Kitchener K. S. (2004). Reflective judgment: theory and research on the development of epistemic assumptions through adulthood. Educ. Psychol. 39, 5–18. doi: 10.1207/s15326985ep3901_2

[ref32] McDonald R. P. (1999). Test theory: A unified treatment. Mahwah, NJ: Lawrence Erlbaum Associates.

[ref33] Nguyen T. V. Kuo C. L. Wang C. Y. Le N. T. Nguyen M. T. T. Chuang Y. H. (2023). Assessment of the psychometric properties of the Vietnamese version of the critical thinking disposition scale. Nurse Educ. Today 127:105848. doi: 10.1016/j.nedt.2023.105848, PMID: 37257290

[ref34] Quinn S. Hogan M. Dwyer C. Finn P. Fogarty E. (2020). Development and validation of the student-educator negotiated critical thinking dispositions scale (SENCTDS). Think. Skills Creat. 38:100710. doi: 10.1016/j.tsc.2020.100710

[ref35] Rammstedt B. (2007). The 10-item big five inventory: norm values and investigation of sociodemographic effects based on a German population representative sample. Eur. J. Psychol. Assess. 23, 193–201. doi: 10.1027/1015-5759.23.3.193

[ref36] Rosnawati R. Kartowagiran B. Jailani J. (2015). A formative assessment model of critical thinking in mathematics learning in junior high school. Res. Eval. Educ. 1, 186–198. doi: 10.21831/reid.v1i2.6472

[ref37] Saltor J. Barberia I. Rodríguez-Ferreiro J. (2023). Thinking disposition, thinking style, and susceptibility to causal illusion predict fake news discriminability. Appl. Cogn. Psychol. 37, 360–368. doi: 10.1002/acp.4008

[ref38] Selda B. A. K. I. R. (2015). Critical thinking dispositions of pre-service teachers. Educ. Res. Rev. 10, 225–233. doi: 10.5897/ERR2014.2021

[ref40] Silva H. Lopes J. Cruz G. Dominguez C. Morais E. (2023). Does university attendance affect students’ critical and creative thinking skills? A longitudinal research with pre-service teaching and psychology undergraduates. High. Educ. Res. Dev. 42, 442–452. doi: 10.1080/07294360.2022.2057448

[ref41] Sk S. Halder S. (2024). Effect of emotional intelligence and critical thinking disposition on resilience of the student in transition to higher education phase. J. Coll. Stud. Retent. Res. Theory Pract. 25, 913–939. doi: 10.1177/15210251211037996

[ref42] Sosu E. M. (2013). The development and psychometric validation of a critical thinking disposition scale. Think. Skills Creat. 9, 107–119. doi: 10.1016/j.tsc.2012.09.002

[ref43] Stupple E. J. Maratos F. A. Elander J. Hunt T. E. Cheung K. Y. Aubeeluck A. V. (2017). Development of the critical thinking toolkit (CriTT): a measure of student attitudes and beliefs about critical thinking. Think. Skills Creat. 23, 91–100. doi: 10.1016/j.tsc.2016.11.007

[ref003] Stanovich K. (2011). Rationality and the reflective mind. Oxford University Press.

[ref44] Suriano R. Plebe A. Acciai A. Fabio R. A. (2025). Student interaction with ChatGPT can promote complex critical thinking skills. Learn. Instr. 95:102011. doi: 10.1016/j.learninstruc.2024.102011

[ref45] Syahfitri J. Firman H. Redjeki S. Sriyati S. (2019). Development and validation of critical thinking disposition test in biology. Int. J. Instr. 12, 381–392. doi: 10.29333/iji.2019.12425a

[ref46] Tavakol M. Dennick R. (2011). Making sense of Cronbach’s alpha. Int. J. Med. Educ. 2, 53–55. doi: 10.5116/ijme.4dfb.8dfd, PMID: 28029643 PMC4205511

[ref47] Teng M. F. Yue M. (2023). Metacognitive writing strategies, critical thinking skills, and academic writing performance: a structural equation modeling approach. Metacogn. Learn. 18, 237–260. doi: 10.1007/s11409-022-09328-5

[ref48] Vincent-Lancrin S. (2023). Fostering and assessing student critical thinking: from theory to teaching practice. Eur. J. Educ. 58, 354–368. doi: 10.1111/ejed.12569

[ref49] Wang X. Sun X. Huang T. He R. Hao W. Zhang L. (2019). Development and validation of the critical thinking disposition inventory for Chinese medical college students (CTDI-M). BMC Med. Educ. 19, 200–214. doi: 10.1186/s12909-019-1593-z, PMID: 31196183 PMC6567520

[ref50] Yeh M. L. (2002). Assessing the reliability and validity of the Chinese version of the California critical thinking disposition inventory. Int. J. Nurs. Stud. 39, 123–132. doi: 10.1016/S0020-7489(01)00019-0, PMID: 11755443

[ref51] Yockey R. D., (2016). Validation study of the critical thinking dispositions scale: a brief report. N. Am. J. Psychol., 18:101. Available online at: https://link.gale.com/apps/doc/A445367736/AONE?u=anon~4bddffd7&sid=googleScholar&xid=2c63454a

[ref52] Zhang L. F. (2003). Contributions of thinking styles to critical thinking dispositions. J. Psychol. 137, 517–544. doi: 10.1080/00223980309600633, PMID: 14992346

[ref53] Zhang Y. Bian Y. Wu H. Tang W. Li Q. (2023). Intuition or rationality: impact of critical thinking dispositions on the cognitive processing of creative information. Think. Skills Creat. 48:101278. doi: 10.1016/j.tsc.2023.101278

[ref54] Zwiers J. Crawford M. (2023). Academic conversations: Classroom talk that fosters critical thinking and content understandings. New York, NY: Routledge.

