# Supplementary material for: Development and psychometric properties of the critical thinking attitude scale in Italian college students
Source: Front Psychol. 2025 Oct 7;16:1599920. doi: 10.3389/fpsyg.2025.1599920 (PMC12537700; doi:10.3389/fpsyg.2025.1599920)
Supplement: Supplementary file 1 [file Data_Sheet_1.PDF]

Online Supplementary Material

Table 1 Items of Critical Thinking Attitude Scale (CTAS)

Items	*Content*	*Source*
1. I know how to think systematically.	Problem solving	Hwang et al. (2010)
2. I am able to think logically.	Problem solving	Hwang et al. (2010)
3. I draw conclusions through precise logical and methodological analyses.	Confidence in Critical Thinking - Systematicity	Stupple et al. (2017)
4. I concentrate continuously when I have to tackle a problem.	Systematic Analysis	Hwang et al. (2010)
5. People say that I make decisions meticulously.	Systematic Analysis	Hwang et al. (2010)
6. I can connect new data with what I already know.	Confidence in Critical Thinking - Systematicity	Stupple et al. (2017)
7. I can read between the lines and find contradictions among the various parts of the text.	Confidence in Critical Thinking - Systematicity	Stupple et al. (2017)
8. I can relate the results of observation to existing theories of knowledge.	Confidence in Critical Thinking - Systematicity	Stupple et al. (2017)
9. Others turn to me to solve their problems.	Systematic Analysis	Hwang et al. (2010)
10. I am curious to get to the bottom of things.	Thinking outside the box - Search for Truth and Openness	Hwang et al. (2010)
11. I always try to delve deeper and understand things thoroughly.	Thinking outside the box - Search for Truth and Openness	Hwang et al. (2010)
12. When something doesn't convince me, I examine all possible alternatives.	Open-mindedness -	Quinn et al. (2020)

	Search for Truth and Openness	
13. I am convinced that we should learn everything we can; you never know when it might come in handy.	Thinking outside the box - Search for Truth and Openness	Hwang et al. (2010)
14. I always try to understand the ideas of others.	Thinking outside the box - Search for Truth and Openness	Hwang et al. (2010)
15. I expect to be able to face the challenges of life.	Attention-Analyticity	Stupple et al. (2017), Quinn et al. (2020)
16. Every evaluation we make must be based on criteria.	Attention-Analyticity	Stupple et al. (2017), Quinn et al. (2020)
17. I can assess the value of each piece of information in a problem.	Thinking within the box-Analyticity	Hwang et al. (2010)
18. I make sure that each piece of information is reliable before putting them together in the problem.	Thinking within the box-Analyticity	Hwang et al. (2010)
19. If I have to work on a problem, I can clear my mind of everything else.	Thinking within the box-Attention - Analyticity	Hwang et al. (2010), Quinn et al. (2020)
20. Some people have the gift of clairvoyance, meaning the ability to know the future.	Paranormal beliefs	Akar-Vural (2005)
21. Extraterrestrials have visited Earth in the past.	Paranormal beliefs	Akar-Vural (2005)
22. The positions of stars and planets can influence people's lives.	Paranormal beliefs	Akar-Vural (2005)
23. I believe curses are effective.	Paranormal beliefs	Akar-Vural (2005)
24. The spirits of deceased individuals can return in certain places and situations.	Paranormal beliefs	Akar-Vural (2005)

25. I know how to use rigorous investigative methods to solve problems.	Critical Openness - Inquisitiveness	Sosu (2013)
26. It would be wonderful to study new things for a lifetime.	Inquisitiveness	Stupple et al. (2017), Quinn et al. (2020)
27. I have a great desire to learn new things.	Critical Openness - Inquisitiveness	Sosu (2013)
28. I am tolerant in welcoming ideas that are different from mine.	Critical Openness-Inquisitiveness	Sosu (2013)
29. I always seek the most reliable sources when I need to understand a problem.	Critical Openness - Inquisitiveness	Sosu (2013)
30. I am curious to investigate even the phenomena that science has not yet explained.	Critical Openness - Inquisitiveness	Sosu (2013)
31. When I analyse information, I try to be objective and honest.	Critical Openness - Inquisitiveness	Sosu (2013)
32. Before making a decision, I try to do everything possible to gather all the information.	Inquisitiveness	Hwang et al. (2010), Stupple et al. (2017)

Table 2 Final Version of Italian Critical Thinking Attitude Scale.

Items
1. So pensare in modo sistematico.
2. Sono in grado di pensare logicamente.
3. Traggo conclusioni attraverso analisi logiche e metodologiche precise
4. Mi concentro in modo continuativo quando devo affrontare un problema.
5. La gente dice che prendo decisioni in modo meticoloso.
6. Riesco a collegare i dati nuovi con ciò che già so.
7. Sono capace di leggere fra le righe e trovo le contraddizioni fra le varie parti del testo.
8. So mettere in relazione i risultati dell'osservazione con le teorie (di conoscenza) esistenti.
9. Altri si rivolgono a me per risolvere i loro problemi.
10. Sono curioso di andare in fondo alle cose.
11. Cerco sempre di approfondire e capire bene le cose.
12. Quando qualcosa non mi convince esamino tutte le alternative possibili.
13. Sono convinto che dobbiamo imparare tutto quello che possiamo, non sai mai quando potrebbe tornare utile.
14. Cerco sempre di riuscire a capire le idee degli altri.
15. Ogni valutazione che facciamo deve essere basata su criteri.
16. So valutare il valore di ogni pezzo di informazione di un problema.
17. Mi assicuro che ogni pezzo di informazione sia attendibile prima di metterli insieme il problema.
18. Se devo lavorare su un problema, riesco a togliermi tutte le altre cose dalla testa.
19. So usare metodi di indagine rigorosi per risolvere i problemi.
20. Sarebbe meraviglioso studiare cose nuove per tutta la vita.
21. Ho una grande voglia di imparare cose nuove.
22. Sono tollerante nell'accogliere anche idee diverse dalle mie.
23. Cerco sempre le fonti più attendibili quando devo capire un problema.
24. Sono curioso di indagare anche i fenomeni che la scienza non ha ancora spiegato.
25. Quando analizzo le informazioni cerco di essere obiettivo e onesto
26. Prima di prendere una decisione cerco di fare tutto il possibile per raccogliere tutte le informazioni.
